# Biological Characteristics of Umbilical Cord Mesenchymal Stem Cells and Its Therapeutic Potential for Hematological Disorders

**DOI:** 10.3389/fcell.2021.570179

**Published:** 2021-05-03

**Authors:** Yufeng Shang, Haotong Guan, Fuling Zhou

**Affiliations:** Department of Hematology, Zhongnan Hospital of Wuhan University, Wuhan, China

**Keywords:** umbilical cord blood, mesenchymal stem cells, hematologic diseases, immunoregulation, hematopoietic microenvironment

## Abstract

Umbilical cord mesenchymal stem cells (UC-MSCs) are a class of multifunctional stem cells isolated and cultured from umbilical cord. They possessed the characteristics of highly self-renewal, multi-directional differentiation potential and low immunogenicity. Its application in the field of tissue engineering and gene therapy has achieved a series of results. Recent studies have confirmed their characteristics of inhibiting tumor cell proliferation and migration to nest of cancer. The ability of UC-MSCs to support hematopoietic microenvironment and suppress immune system suggests that they can improve engraftment after hematopoietic stem cell transplantation, which shows great potential in treatment of hematologic diseases. This review will focus on the latest advances in biological characteristics and mechanism of UC-MSCs in treatment of hematological diseases.

## Introduction

Mesenchymal stem cells (MSCs) are multipotent stem cells that originate in mesoderm at early development. MSCs were first discovered in bone marrow and later confirmed to be isolated from a variety of human tissues, such as adipose tissue, nervous tissue, umbilical cord and amniotic fluid (Kobolak et al., 2016; Zhan et al., 2019). Bone marrow MSCs (BM-MSCs) is extremely low while there is a high possibility of virus contamination. Moreover, the number of stem cells, the ability to expand and differentiate significantly decreased with age, which limits their clinical application (Kern et al., 2006). Subsequent studies have shown that human umbilical cords contain large amounts of MSCs (Kern et al., 2006). Traditionally, umbilical cord tissue is regarded as a waste product after birth, so there is no ethical controversy to isolate MSCs from umbilical cord tissue compared with obtaining MSCs from bone marrow. The morphological, immunophenotype, proliferation, multi-directional differentiation and the ability to promote differentiation of hematopoietic stem cells (HSCs) in UC-MSCs are mostly similar to those BM-MSCs (Thaweesapphithak et al., 2019), but UC-MSCs have higher proliferative capacity and lower human leukocyte antigen (HLA)-ABC and HLA-DR expression than BM-MSCs. In addition, UC-MSCs with a wide variety of stem cells are well-sourced, easy to collect and preserve. UC-MSCs are expected to be an ideal alternative source for BM-MSCs. UC-MSCs play a unique role in reducing incidence and severity of graft versus host disease (GVHD) which occurs in more than 50% of patients undergoing hematopoietic stem cell transplantation (HSCT) (Zhao et al., 2019). Besides, based on their migratory capability toward cancer cells, many reports have proposed UC-MSCs as cell therapy to target tumors and to locally deliver anti-cancer molecules (Bajetto et al., 2017). However, various aspects of research on UC-MSCs are still in their infancy. The present review briefly overviews biological characteristics of UC-MSCs, current research advances and their application in treatment of hematological diseases.

## Biological Characteristics of UC-MSCs

### Immunological Properties of UC-MSCs

UC-MSCs are mainly located in subcortical endothelium of umbilical cord, perivascular region and Wharton’s Jelly (WJ), which was mainly composed of sponge-like structure woven with collagen fibers, proteoglycans and embedded stromal cells (Watson et al., 2015). Flow cytometry analysis of UC-MSCs from WJ showed that CD24 and CD108 were highly expressed, while the expression of fibroblast-specific markers (FAP and FSP) and dermal fibroblast marker CD40 were very low. This suggests that WJ is a region rich in MSCs (Subramanian et al., 2015). UC-MSCs could express characteristic markers of various cells. In addition to expressing MSCs markers (CD105, CD90, CD73), UC-MSCs also expressed adhesion molecule markers (CD54, CD13, CD29, CD44). Low or no expression of CD31, CD14, CD34, CD45 and other hematopoietic stem cell-associated surface antigens; no expression immune response-related antigens involved in T lymphocyte activation, such as CD80, CD86 and CD40, CD40L and major histocompatibility complex (MHC) class II antigen HLA-DR (Wang et al., 2014; Stefanska et al., 2020). The expression of CD106 and HLA-ABC in UC-MSCs were significantly lower than that of BM-derived cells, which result in low immunogenicity of UC-MSCs (Lu et al., 2006).

### Proliferation and Differentiation Potential of UC-MSCs

WJ-MSCs displayed the highest proliferation rate with three to four times higher than that of adipose tissue (AT-) and BM-MSCs (Amable et al., 2014; Kim et al., 2018). The Mennan team’s report indicated that there was no significant difference in proliferation rate no matter what compartment UC-MSCs come from, and the average doubling time of UC-MSCs between P0 and P3 was 2–3 days, significantly faster than BM-MSCs (Mennan et al., 2013). UC-MSCs have the ability of multi-directional differentiation and have potential to differentiate into bone, adipose, cartilage and other tissues (Kfoury and Scadden, 2015; Richardson et al., 2016; Liu et al., 2018; Zhang et al., 2018; Shen et al., 2019; Yea et al., 2020), thus they can be used to repair various tissues and organs, and are ideal seed cells in the field of regenerative medicine (Wang et al., 2020). Studies have shown that when body tissues suffer from ischemia-anoxia injury or chronic inflammation, the damaged tissue released chemokines, mobilized and guided the migration of MSCs to the injury site, and further induced differentiation into different types of cells (Kidd et al., 2010). *In vitro* culture, UC-MSCs can be “trans-differentiated” under certain conditions to become mesoderm cells such as osteoblasts (Xue et al., 2018) and cardiomyocytes (Wu et al., 2018), endothelial cells (Motawea et al., 2020), and also can differentiate into neurons in the ectoderm, hepatocytes (Zhou et al., 2017), pancreatic cells (Van Pham et al., 2014) in endoderm between germ layer. The low immunogenicity, high proliferation and differentiation potential of UC-MSCs make them seed cells for cell replacement therapy.

## The Mechanism of UC-MSCs Applied to Hematologic Diseases

### UC-MSCs Support Hematopoiesis and Promote Engraftment and Expansion of HSCs

UC-MSCs have been shown to support HSCs growth *in vitro* and *in vivo* (Fan and Zhang, 2011; Fajardo-Orduna et al., 2017). MSCs play an important role in regulating hematopoiesis and supporting engraftment and expansion of HSCs by secreting various adhesion molecules, cytokines and cell-cell contact interactions (Friedman et al., 2007; Le Blanc et al., 2007). When UC-MSC and HSCs were co-cultured *in vitro*, MSC highly expressed hematopoietic related factors promoting the growth of HSCs, and no significant difference was observed in colony-forming cells between the CD34 + cells/UC-MSC and CD34 + cells/BM-MSC co-cultures, which indicated that as a source of MSCs for cell therapies, UC was an excellent alternative to BM (Lu et al., 2006). Cytokine spectroscopy studies showed that UC-MSCs, like BM-MSC, expressed stem cell factor (SCF), leukemia inhibitory factor (LIF), macrophage-colony stimulating factor (M-CSF), FMS-like tyrosine kinase 3 (FLT3), interleukin-6 (IL-6) and stromal cell derived factor-1 (SDF-1) (Lu et al., 2006). Moreover, UC-MSC also produced cytokine such as granulocyte colony-stimulation factor (G-CSF) and granulocyte-macrophage colony stimulating factor (GM-CSF), which were not found in BM-MSC (Lu et al., 2006; Friedman et al., 2007). These cytokines are associated with HSCs proliferation, survival and differentiation. In a study by Bakhshi et al. (2010), UC-MSCs supported the growth of CD34^+^ cord blood cells in a long-term culture-initiating cell assay. UC-MSCs also increased homing and improved migration efficiency of UCB CD34^+^ cells to bone marrow and spleen by expressing a high level of homing adhesion molecules (Hao et al., 2009). This suggested the potential therapeutic application of UC-MSCs to provide stromal support structure for the long-term culture of HSCs as well as the possibility of co-transplantation of genetically identical, HLA-matched, or unmatched cord blood HSCs (Bakhshi et al., 2010; Lin et al., 2020). There are some reports that the expression level of SDF-1 in UC-MSC is lower than that of MSC derived from bone marrow or adult tissue (Fonseka et al., 2013). SDF-1 could inhibit apoptosis of stem cells and was associated with hematopoietic stem cell proliferation and survival, which indicated that UC-MSCs have weak hematopoietic supportive capacity than BM-MSCs. Overexpression of SCF and SDF-1 by UC-MSCs in coculture system has efficient effect on the expansion of UCB-HSCs (Ajami et al., 2019).

### UC-MSCs Inhibit Proliferation of Hematological Malignancies Cell

**FIGURE 1:**
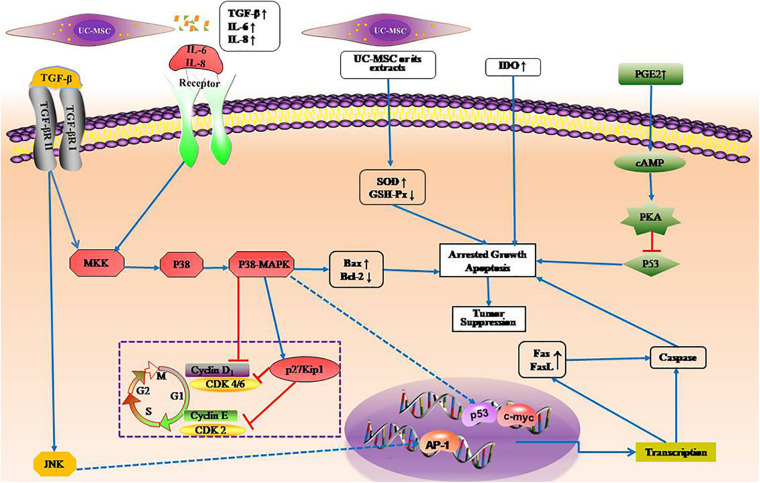
Possible signal pathways of umbilical cord mesenchymal stem cells to hematological tumors. TGFβ, IL6, and IL8 not only arrest cell cycle in Go or G1 phase inhibiting the proliferation of leukemia cells, but also promote apoptosis of leukemia cells. UC-MSCs or their secretions induce lymphoma cell apoptosis by oxidative stress pathway by enhancing SOD activity and decreasing GSH-Px activity. Forced expression of IDO enhance proliferation inhibitory effect on leukemia cells. PGE2 promoting leukemogenesis by inhibiting activity of p53 via cAMP-PKA pathway. SOD, superoxide dismutase; GSH-Px, glutathione peroxidase; IDO, indoleamine 2,3-dioxygenase.

UC-MSCs participate in immune responses to tumor cells through various pathways. UC-MSCs do not induce tumorigenesis (Gauthaman et al., 2012)and also has anti-cancer properties (Wang et al., 2018; Li et al., 2019). The anti-cancer properties of UC-MSCs may be related to their low immunogenicity, the ability to secrete cytokines and transdifferentiate. Tian et al. (2010) believed that UC-MSCs could activate p38MAPK signaling pathway in HL-60 and K562 leukemia tumor cells, which has a strong inhibitory effect on the proliferation of these leukemia cells. This inhibition is achieved by arresting the cell cycle rather than by inducing apoptosis, indicating that UC-MSCs have the ability to induce tumor dormancy. There are other studies supporting the conclusion that UC-MSCs arrested tumor cells in specific phases of cell cycle, increased apoptosis and attenuated the migratory abilities of tumor cells (Yuan et al., 2018). In addition, Ramasamy et al. (2007) found that human bone marrow and cord blood-derived MSCs can down-regulate the expression of cyclin D2 and CDK4 with the inhibition of p27kip1 to arrest leukemia cells in Go or G1 phase to inhibit the proliferation of leukemia cells. In the subsequent study, they detected cytokines in co-culture supernatant of UC-MSCs and leukemia cell lines, and found that the expression of transforming growth factor-β1(TGF-β1), IL-6, and IL-8 was significantly increased, to conclude that cytokines secreted by MSCs are related to their inhibition of leukemic cell proliferation (Fonseka et al., 2013). Besides, UC-MSCs or their secretions may induce lymphoma cell apoptosis by oxidative stress pathway (Lin et al., 2016). It was demonstrated that UC-MSCs induce T lymphocyte apoptosis and cell cycle arrest by expressing abundant IDO (Li et al., 2016). UC-MSC also inhibits proliferation of malignant cells as a member of immune cells. UC-MSCs may phagocytose some components of apoptotic lymphoma cells, so it could be speculated that UC-MSCs have the function of antigen presenting cells (Lin et al., 2014). In addition, UC-MSCs also exert anti-tumor effect as a carriers for gene or drug therapy (Zhang X. et al., 2017; Cao et al., 2018; Yuan L. et al., 2019). However, it is worth noting that some studies have shown that MSCs has the possibility to promote tumor progression (Li et al., 2018; Zhou et al., 2019). For example, PGE2 secreted by MSCs, can inhibit activity of p53, thereby promoting leukemogenesis and protecting against therapy-induced leukemic cell death through activation of cAMP-PKA signaling in BCP-ALL blasts (Naderi et al., 2015). The possible signaling pathways of UC-MSCs to malignant hematological cells is shown in Figure 1.

### UC-MSCs Inhibit Immune System

**FIGURE 2:**
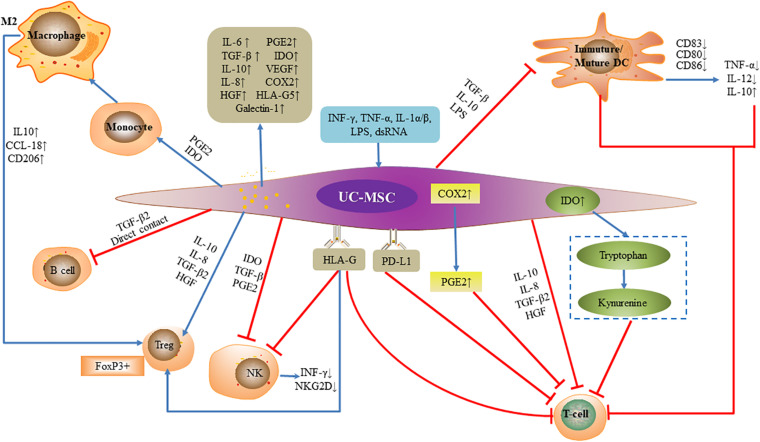
Immunomodulatory function of umbilical cord mesenchymal stem cells. Under the license of INF-γ, TNF-α, IL-1α/β, LPS, and dsRNA, umbilical cord mesenchymal stem cells secrete inflammatory factors and alter the expression of surface antigen to exert effects. Umbilical cord mesenchymal stem cells inhibit the proliferation and cytotoxicity of activated T lymphocytes, and secretion of inflammatory factors, inhibit B lymphocytes and secretion of immunoglobulins, inhibit NK cell killing activity and proliferation, and inhibit differentiation of monocytes into DCs achieving tolerogenic phenotype. Umbilical cord mesenchymal stem cells promote regulatory T cells.

The immunosuppressive function of MSCs is powerful and has varying degrees of effects on almost the entire immune network (Figure 2). Two different mechanisms, either cell contact-dependent or -independent, have been advanced to explain this immunosuppression (Yang et al., 2017; Zhang H. et al., 2017; Min et al., 2020). The main way is to inhibit the proliferation of activated T lymphocytes and secretion of inflammatory factors, inhibit the proliferation of B lymphocytes and secretion of immunoglobulins, inhibit the killing activity of NK cells, and inhibit the differentiation of monocytes into dendritic cells (DCs) (Dabrowski et al., 2017; Xie et al., 2020).

UC-MSCs can release secretory soluble media such as IL-6, IL-8, TGF-β, IDO, vascular endothelial growth factor (VEGF) and cyclooxygenase-2 (COX-2), and prostaglandin E2 (PGE2), hepatocyte growth factor (HGF), Galectin-1 and HLA-G5 as an effective factor for immunosuppression (Amable et al., 2014; Ding et al., 2016; Abolhasani et al., 2018; Zhang et al., 2019). The secretion of inhibitory cytokines such as IL-10, IL-6, IL-8, TGF-β2, and HGF inhibit Th17 cells and stimulate Treg cells, furthermore, UC-MSCs can also inhibit the proliferation of activated T cells by secreting IDO and PGE2, as well as upregulating the expression of programmed death ligand 1 (PD-L1) (Deng et al., 2016; Wang D. et al., 2017; Kim et al., 2018; Song et al., 2020). When the supernatants and cells produced by co-culture of UC-MSCs and phytohemagglutinin (PHA)-stimulated peripheral blood mononuclear cells (PBMCs) were compared with those of PHA-stimulated PBMCs without UC-MSCs, IFN-γ was significant down-regulation while PGE2, TGF-β1, and IL-10 were greatly up-regulated, meanwhile CD4^(+)^CD25(high)CD45RA^(+)^Tregs increased. This suggests that UC-MSCs can inhibit PHA- stimulated lymphocyte proliferation to exert immunosuppressive functions by regulating T cell differentiation and increasing the production of associated suppressive cytokines (Yang et al., 2016).

Upregulation of COX-2 expression by UC-MSCs also contributes to the production of PGE2, which directly inhibits T cell proliferation by increasing intracellular cAMP levels (van der Pouw Kraan et al., 1995; Kim et al., 2018). MSCs also inhibit T cell proliferation by eliminating tryptophan from the environment, and inhibit IFN-γ-activated T cells by IDO, which not only affects T cell proliferation but also reduces endogenous IFN-γ production by CD8 + T cells (Hong et al., 2016; Li et al., 2016). UC-MSCs also express non-classical HLA class I molecule HLA-G (Ding et al., 2015). HLA-G regulates multiple T cell subtypes: it not only inhibits T cell proliferation, but also attenuates cytotoxicity and cell-mediated cytolysis (Ding et al., 2016; Szaraz et al., 2016). In addition, HLA-G molecules can induce CD4^+^ CD25high FOXP3^+^ regulatory T cells, and inhibit the cytolytic function of NK cells to adjacent cells and down-regulate the secretion of IFN-γ by NK cells (Selmani et al., 2010). UC-MSCs also induce DCs to acquire tolerogenic phenotype through IL-6-mediated upregulation of suppressor of cytokine signaling 1 (SOCS1), which blocked DC maturation by impairing the TLR4 signaling leading to the immune tolerance. MSC-DCs showed a tolerogenic characteristics, the expression of co-stimulatory factors and the capacity to induce CD3 + T cell proliferation and inflammatory factor secretion were significantly decreased, and the production of inhibitory cytokine IL-10 and the ability to induce Treg cells and Th2 responses increased (Deng et al., 2014; Yuan X. et al., 2019).

## Application of UC-MSCs in Hematologic Diseases

### Rationality of UC-MSCs Combined Hematopoietic Stem Cell Transplantation

Hematological diseases are a series of heterogeneous diseases associated with abnormal blood changes that originate from the hematopoietic system or affect the hematopoietic system. Autologous or allogeneic HSCT are critical therapy tools to treat malignant and non-malignant hematological diseases (Bair et al., 2020; Mallhi et al., 2020). In order to completely clear immune system of recipient before HSCT, it is necessary to use high-dose chemotherapy drugs for pretreatment. However, the negative effects of bone marrow hematopoietic microenvironment damage and delayed immune reconstitution may lead to poor stem cell engraftment, and increase the rate of postoperative infection and recurrence (Bair et al., 2020; Mallhi et al., 2020). A series of animal and clinical trials showed that HSCT or cord blood transplantation (CBT) combined with infusion of UC-MSCs is able to promote hematopoietic engraftment and reduce the occurrence GVHD (Liu et al., 2020c; Yang et al., 2020). It was reported that UC-MSCs promote myeloid-derived suppressor cell proliferation by secreting HLA-G to reduce acute GVHD after HSCT (Yang et al., 2020). UC-MSCs produce an immunosuppressive isoform of HLA-1, do not express HLA-DR and lack costimulatory signaling systems and immune response-related surface antigens such as CD40, CD40L, CD80, and CD86, which indicate that UC-MSCs can be tolerated in allogeneic transplantation (Wang et al., 2014). There were no reports of any toxicity or side effects when UC-MSCs were injected into laboratory animals in pre-clinical trials (Fan and Zhang, 2011), or were used to treat aplastic anemia (Xu et al., 2018; Liu et al., 2020c), leukemia (Kang-Hsi et al., 2013; Wu Y. et al., 2013) in clinical studies. This indicates that it is safe enough to use allogeneic MSCs after the HLA molecule is detected.

### The Ability of UC-MSCs to Treat Graft Versus Host Response

GVHD is an immunological disorder that donor immune cells attack healthy recipient tissues, including gastrointestinal tract, liver, skin, and lungs, posing a threat to life (Nassereddine et al., 2017). Gao et al. (2016) have reported that repeated infusion of MSCs after patients’ allogeneic hematopoietic stem cell transplantation (Allo-HSCT) can increase Treg cell, decrease memory B lymphocytes and NK cells, and change the ratio of Th1:Th2 cells, leading to the acquisition of immune tolerance, and then inhibit GVHD, improve transplantation survival rate. No serious side effects were found after infusion of 3/6–5/6 HLA-matched UC-MSCs, and the engraftment of neutrophils and platelets was faster in patients who co-transplanted with cord blood and UC-MSCs than in patients who received CBT alone. This suggests that UC-MSCs enhance engraftment after CBT (Wu K.H. et al., 2013). Peng et al. (2015) treated chronic GVHD with MSCs in 23 patients, and 20 patients were found to have complete or partial remission after 1 year of follow-up. In these patients who achieved MSCs treatment, the number of IL-10-producing CD5 + regulatory B cells (Bregs) significantly increased, and CD5 + B cells showed increased IL-10 expression, which was associated with reduced inflammatory cytokine production by T cells. The mechanism was that MSCs could promote the survival and proliferation of CD5 + Bregs, and IDO partially participates in the MSC-mediated effects on Bregs. It was also reported by other study that UC-MSCs boosted the numbers of CD5^(+)^ B cells and IL-10-producing CD5^(+)^ Bregs, and corrected Treg/Th17/Th1 imbalances (Chao et al., 2016). Yang et al. (2020) believed that UC-MSCs promote myeloid-derived suppressor cell proliferation by secreting HLA-G to reduce acute GVHD after HSCT. In a mouse model of allo-HSCT, after intravenously administered exosomes of UC-MSCs to recipient mice, the frequencies and absolute numbers of CD3^+^ CD8^+^ T cells decreased, the proportion of CD3^+^ CD4^+^ and CD3^+^ CD8^+^ T cells increased, meanwhile, the serum levels of IL-2, TNF-α, and IFN-γ reduced, but IL-10 increased. In this way, the occurrence of acute GVHD is prevented (Wang et al., 2016). These findings suggest that UC-MSCs are ideal alternative in the prophylaxis of GVHD after allo-HSCT (Table 1). University of Kansas Medical Center is conducting clinical trial about the evaluation of UC-MSCs for the treatment of acute GVHD (NCT03158896).

**TABLE 1:** Application of UC-MSCs in hematologic diseases.

**Type of disease**	**The Role of UC-MSCs**	**Possible mechanisms of therapeutic effect by MSCs**
Malignant hematological diseases: non-Hodgkin’s lymphoma	As cell carriers (such as MSC-Tandab (CD3/CD19) and scFvCD20-sTRAIL) to treat hematological malignancies	• Tumor tropism • Low immunogenicity • Easy expansion
Malignant and non-malignant hematological diseases	Co-transplantation with HSCT to reduce GVHD and promote hematopoietic engraftment	• HLA-1^(+)^, HLA-DR^(–)^, CD40^(–)^, CD40L^(–)^, CD80^(–)^, CD86^(–^^)^ • Treg↑, CD5 + Bregs↑, Bm cells↓, NK cells↓, CD4 + :CD8 + T cells ↑, Th1:Th2 cells↓, IL-2↓, TNF-α↓, IFN-γ↓, IL-10 ↑ • Produce cytokine, such as G-CSF, GM-CSF
SAA	Improve survival and quality of life in patients with SAA by co-transplantation with HSCT or PBSCs	• Accelerated hematopoietic reconstitution • Reduced incidence of severe GVHD and transplant-related mortality
APL	The potential to treat APL	• Activating MEK/ERK signaling to induce granulocyte differentiation of APL-derived NB4 cell lines and primary APL cells
ITP	The potential to treat ITP	• Regulate secretion of PAIgG • Inhibited the proliferation of platelet-reactive T helper cell • Promoting platelet production • Reversed the dysfunctions of megakaryocytes

*APL, acute promyelocytic leukemia; Bm cells, memory B cells; G-CSF, granulocyte colony-stimulation factor; GM-CSF, granulocyte-macrophage colony stimulating factor; GVHD, graft versus host disease; HLA, human leukocyte antigen; HSCT, hematopoietic stem cell transplantation; ITP, Immune thrombocytopenic; MSC, mesenchymal stem cells; PAIgG, IgG antiplatelet antibody; SAA, severe aplastic anemia; scFvCD20-sTRAIL, contains a CD20-specific single chain Fv antibody fragment (scFv) and a soluble tumor necrosis factor related apoptosis-inducing ligand (sTRAIL); Tandab (CD3/CD19), tetravalent bispecific tandem diabody with two binding sites for CD3 and two for CD19.*

### The Ability of UC-MSCs to Treat Aplastic Anemia

Severe aplastic anemia (SAA) is a life-threatening condition characterized by bone marrow hypoplasia and pancytopenia (Bacigalupo, 2017). Allo-HSCT is recommended as the first-line treatment in young patients with an available matched related donor (MRD) and as the second-line treatment in older patients who failed immunosuppressive therapy (IST) (Killick et al., 2016; Bacigalupo, 2017). IST with a combination of antithymocyte globulin (ATG) and cyclosporin A (CsA) is the preferred first-line treatment for patients without an MRD and older patients (Young, 2018). However, about 30–40% of the patients will eventually relapse or become refractory to IST; those unresponsive to initial IST will be considered for transplantation using an alternative donor (Liu et al., 2020a). However, transplantation in patients with acquired SAA often fail to recover, and graft failure and rejection remain a common fatal risk for patients. When co-transplantation of haplo-HSCT and the donor-derived UC-MSCs for SAA patients, it had fewer complications, accelerated hematopoietic reconstitution, reduced incidence of severe GVHD and transplant-related mortality (Wu et al., 2014; Liu et al., 2020b). In addition, several studies have shown that co-transplantation of haplo-HSCT and UC-MSCs from third-party donors has yielded favorable results for SAA patients: overall survival and GVHD-free are improved, which demonstrate a feasible choice for SAA patients without matched donors in improving donor engraftment and reducing severe GVHD (Wu et al., 2011; Liu et al., 2020b). Clinical data from co-transplantation of unrelated donor peripheral blood stem cell (PBSCs) and UC-MSCs for young patients with refractory SAA also showed similar results (Fu et al., 2013). Consequently, co-transplantation with UC-MSCs can greatly improve survival and quality of life in patients with SAA (Table 1).

### The Ability of UC-MSCs to Treat Hematological Malignancies

Co-transplantation of haplo-HSCT with third-party donor-derived UC-MSCs can also be used in refractory/recurrent hematologic malignancies to improve donor engraftment and reduce GVHD (Wu Y. et al., 2013; Zhao et al., 2019). MSCs may be a double-edged sword. Studies have shown that patients with hematologic malignancies undergoing co-transplantation of BM-MSC and HSC after chemotherapy have a higher tumor recurrence rate than patients who only received HSC transplantation (Ning et al., 2008). Other study demonstrated that use of UC-MSCs tended to reduce relapse, whereas use of BM-MSCs tended to increase relapse (Zhao et al., 2019). Those findings suggested that BM-MSCs were not a good candidate cell type for GVHD prophylaxis in comparison with UC-MSCs. But it was also worth noting that UC-MSCs was proved to promote the proliferation of Raji cells *in vitro* (Li et al., 2018). MSC co-transplantation in allo-HSCT should be applied in the non-malignant hematopoietic diseases other than malignant hematopoietic diseases at present (Ning et al., 2008). We therefore need to seriously study the relationship between MSCs infusion and disease recurrence, and take corresponding measures to improve the safety and effectiveness of MSCs for the treatment of hematological malignancies. The use of MSCs must be handled with extreme caution before a large-scale clinical trial is performed.

Acute promyelocytic leukemia (APL) is characterized by accumulation of cells blocked in promyelocytic phase. The treatment outcome of APL has dramatically improved over the past three decades following the development of novel agents, such as all-trans retinoic acid (ATRA), which induce terminal differentiation of APL cells into mature granulocytes (Sanz et al., 2019). It has been reported that UC-MSCs can exert similar effects, possibly by activating the MEK/ERK signaling to induce granulocyte differentiation of APL-derived NB4 cell lines as well as primary APL cells. These results demonstrate a stimulatory effect of MSCs on the differentiation of APL cells and bring a new insight into the interaction between MSCs and leukemic cells. It suggest that UC-MSCs/ATRA combination could be used as a novel therapeutic strategy for APL patients (Chen et al., 2013; Table 1).

### UC-MSCs as Cell Carriers to Treat Hematological Malignancies

Multiple chemotherapeutic and molecular targeted agents are available for the treatment of hematological malignancies (Deshantri et al., 2018; Testa et al., 2019). However, only a subset of patients will achieve long-term remission or complete cure of the disease, which is at least partly due to the lack of specificity of these agents for the disease site and their short biological half-lives. Lack of specificity results in off-target adverse effects, and high doses and frequent dosing to maintain the therapeutic levels further increase adverse effects (Egusquiaguirre et al., 2012; Deshantri et al., 2018). Drug delivery systems could be essential to retain the active substance in the circulation and deliver it to the malignant cells (Galderisi et al., 2010; Deshantri et al., 2018). UC-MSCs are an attractive candidate as cell carriers for cell-based therapy to treat malignant diseases because they have ability to migrate to tumor sites and track micrometastasis (Galderisi et al., 2010; Bajetto et al., 2017). The tumor tropism, low immunogenicity and easy expansion with a consistent collection process make MSCs an ideal delivery vehicle for anti-tumor factors (Galderisi et al., 2010). Accurate treatment for anti-tumor can be achieved owing to the delivery vehicles allowing specific tumor targeting and controlled release strategies (Table 1; Jing et al., 2014; Shou et al., 2016).

Bispecific T cell engaging antibody, exhibits high clinical response rates in patients with relapsed or refractory B-precursor acute lymphoblastic leukemia (B-ALL) and B cell non-Hodgkin’s lymphoma (B-NHL), but it still has some limitations because of its short half-life. When human UC-MSCs were genetically modified to constitutively secrete Tandab (CD3/CD19) (MSC-Tandab), a tetravalent bispecific tandem diabody with two binding sites for CD3 and two for CD19, MSC-Tandab was functional with high-binding capability both for CD3-positive cells and CD19-positive cells and can induce specific lysis of CD19-positive cell lines in the presence of T cells. Luciferase-labeled MSCs could selectively migrate to tumor site in BALB/c nude mouse model to significantly inhibit the tumor growth (Zhang X. et al., 2017). Xiong et al. designed a promising double-target therapeutic system for NHL therapy. In this system, a novel secreted fusion protein scFvCD20-sTRAIL, which contains a CD20-specific single chain Fv antibody fragment (scFv) and a soluble tumor necrosis factor related apoptosis-inducing ligand (sTRAIL), was expressed in UC-MSCs. The scFvCD20-sTRAIL fusion protein could inhibit cell proliferation and increase cellular apoptosis through both extrinsic and intrinsic apoptosis signaling pathways. UC-MSCs selectively migrated to the tumor site after 24 h of intravenous injection and tumor growth was significantly inhibited with well tolerated and no toxic reactions occurred (Yan et al., 2013).

### The Ability of UC-MSCs to Treat Immune Thrombocytopenic Purpura

Immune thrombocytopenic (ITP) is an autoimmune disease characterized by antibody-mediated platelet destruction and variable reduced platelet production (Tinazzi et al., 2020). Other immune dysfunctions also participate in ITP pathogenesis, including numeric and functional defects in suppressor T (Ts) cells and immune-regulation abnormalities in MSCs (Li et al., 2020). Firstline treatments include steroids and intravenous immunoglobulins, and thrombopoietin receptor agonists (TPO-RAs) and rituximab were also frequently used before the chronic phase (Moulis et al., 2017; Mahevas et al., 2020). Recent studies conclude that defects of BM-MSCs in Ts cell induction are involved in ITP pathogenesis, and exogenous UC-MSCs may be useful for ITP therapy (Li et al., 2020). When UC-MSCs were co-cultured with splenocytes isolated from patients with ITP *in vitro*, the results showed that UC-MSCs could stimulate the spontaneous secretion of anti-platelet antibody IgG (IgG antiplatelet antibody, PAIgG). However, under platelet-inducing conditions, UC-MSCs inhibited the production of PAIgG at a low ratio of UC-MSC to splenocytes. In addition, UC-MSC inhibited the proliferation of platelet-reactive T helper cell in a dose-dependent manner. Therefore, UC-MSCs can regulate secretion of antiplatelet antibodies *in vitro* (Qiu et al., 2008). Not just *in vitro*, UC-MSCs has been used to treat ITP *in vivo* (Ma et al., 2012; Wang X. et al., 2017). UC-MSCs obviously reversed the dysfunctions of megakaryocytes by promoting platelet production and decreasing the number of living megakaryocytes as well as early apoptosis. The level of thrombopoietin and platelet numbers was also significantly increased to alleviate refractory ITP. These findings suggested that UC-MSCs transplantation might be a potential therapy for ITP (Table 1). The clinical trial of UC-MSCs for treatment of refractory ITP is recruiting (NCT04014166). However, the clinical application of UC-MSCs in treatment of ITP is not yet mature, and its specific regulatory mechanisms and potential immunotherapeutic value need further study.

## Conclusion

The low immunogenicity and immunoregulatory activity of UC-MSCs made it particularly attractive for therapeutic exploitation, which provided the footstone of inhibiting the growth of tumor cells, improving the complications after hematopoietic stem cell transplantation, promoting hematopoiesis for hematologic diseases. Although the research on UC-MSCs has achieved encouraging results, there are still some problems to be solved: The MSCs isolated by the current method have no specific phenotype, leading to a group of mixed cells with different characteristics. It is necessary to explore appropriate cell surface molecular markers for separation and purification, find means to control their growth and differentiation, and investigate whether there is a sign of malignant tendency and malignant transformation after long-term cultivation (Jeschke, 2011). Besides, the MSCs alone have no immunosuppressive effect, but have to be licensed by cytokines such as TNF-α and IFN-γ, and UC-MSCs do not spontaneously form medullary cavities like BM-MSCs through vascularized cartilage intermediates *in vivo* (English, 2013). It will be the main research direction to improve the isolation and culture of MSCs, clarify the mechanisms in immunosuppression and further explore differentiation potential of UC-MSCs. It is believed that with the deepening of research, the application prospect of UC-MSCs in hematologic diseases will become much broader.

## Author Contributions

FZ contributed to the conception, logic and revision of the review. YS and HG contributed to the writing and drafting of the manuscript. All authors contributed to the article and approved the submitted version.

## Conflict of Interest

The authors declare that the research was conducted in the absence of any commercial or financial relationships that could be construed as a potential conflict of interest.
